# A structured collaborative approach to intervention design using a modified intervention mapping approach: a case study using the Management and Interventions for Asthma (MIA) project for South Asian children

**DOI:** 10.1186/s12874-020-01148-y

**Published:** 2020-11-02

**Authors:** Monica Lakhanpaul, Lorraine Culley, Noelle Robertson, Emma C. Alexander, Deborah Bird, Nicky Hudson, Narynder Johal, Melanie McFeeters, Charlotte Hamlyn-Williams, Logan Manikam, Yebeen Ysabelle Boo, Maya Lakhanpaul, Mark R. D. Johnson

**Affiliations:** 1 Population, Policy and Practice, UCL Great Ormond Street Institute of Child Health, 30 Guildford Street, London, WC1N 1EH UK; 2 Whittington Health NHS Trust, London, UK; 3 School of Applied Social Sciences, De Montfort University, The Gateway, Leicester, LE1 9BH UK; 4 Clinical Psychology, Department of Neuroscience, Psychology and Behaviour, University of Leicester, Lancaster Road, Leicester, LE1 7HA UK; 5 Paediatric Liver, GI and Nutrition Centre and Mowatlabs, King’s College Hospital, London, SE5 9RS UK; 6 Aceso Global Health Consultants Ltd., 3 Abbey Terrace, London, SE2 9EY UK; 7 Child Development Team, Ealing Services for Children with Additional Needs, West London NHS Trust, Carmelita House, 21-22 The Mall, Ealing, W5 2PJ UK; 8 Parent representative, Leicester, UK; 9 Specialised Commissioning, NHS England and NHS Improvement, Midlands Region, Fosse House, 6 Smith Way, Grove Park, Enderby, Leicester, LE19 1SX UK; 10 Center for Health Services and Clinical Research, University of Hertfordshire, College Lane Campus, Hatfield, AL10 9AB UK; 11 UCL Institute of Epidemiology & Healthcare, 1 – 19 Torrington Place, London, WC1E 7HB UK; 12 Nuffield Department of Population Health, University of Oxford Richard Doll Building, Old Road Campus, Headington, Oxford, OX3 7LF UK; 13 Faculty of Biology, Medicine and Health, University of Manchester, Oxford Road, Manchester, M13 9PL UK; 14 Faculty of Health and Life Sciences, Mary Seacole Research Centre, De Montfort University, The Gateway, Leicester, LE1 9BH UK

**Keywords:** Intervention mapping, Minority ethnic research, Asthma management, Collaborative, Tailored, Community based participatory research

## Abstract

**Background:**

To describe how using a combined approach of community-based participatory research and intervention mapping principles could inform the development of a tailored complex intervention to improve management of asthma for South Asian (SA) children; Management and Interventions for Asthma (MIA) study.

**Methods:**

A qualitative study using interviews, focus groups, workshops, and modified intervention mapping procedures to develop an intervention planning framework in an urban community setting in Leicester, UK. The modified form of intervention mapping (IM) included: systematic evidence synthesis; community study; families and healthcare professionals study; and development of potential collaborative intervention strategies. Participants in the community study were 63 SA community members and 12 key informants; in-depth semi-structured interviews involved 30 SA families, 14 White British (WB) families and 37 Healthcare Professionals (HCPs) treating SA children living with asthma; prioritisation workshops involved 145 SA, 6 WB and 37 HCP participants; 30 participants in finalisation workshops.

**Results:**

Two key principles were utilised throughout the development of the intervention; community-based participatory research (CBPR) principles and intervention mapping (IM) procedures. The CBPR approach allowed close engagement with stakeholders and generated valuable knowledge to inform intervention development. It accounted for diverse perceptions and experiences with regard to asthma and recognised the priorities of patients and their families/caregivers for service improvement. The ‘ACT on Asthma’ programme was devised, comprising four arms of an intervention strategy: education and training, clinical support, advice centre and raising awareness, to be co-ordinated by a central team.

**Conclusions:**

The modified IM principles utilised in this study were systematic and informed by theory. The combined IM and participatory approach could be considered when tailoring interventions for other clinical problems within diverse communities. The IM approach to intervention development was however resource intensive. Working in meaningful collaboration with minority communities requires specific resources and a culturally competent methodology.

**Supplementary information:**

**Supplementary information** accompanies this paper at 10.1186/s12874-020-01148-y.

## Background

A 2019 report from the Nuffield Trust found that health outcomes for young people with long-term conditions in the UK were among the worst compared to 19 similarly high-income countries [1]. Children from minority ethnic backgrounds are particularly disadvantaged with regards to long-term conditions in terms of morbidity and mortality [2]. The need to reduce these inequalities is widely recognised both by UK governments [3, 4] and health professionals [5].

Asthma is a heterogeneous disease, usually characterised by chronic airway inflammation; the symptoms include one or more respiratory symptoms such as wheeze, shortness of breath, chest tightness and cough [6]. It is a global health problem affecting all age groups; however, it is more prevalent in childhood. Existing guidelines, such as the Global Initiative for Asthma (GIA) guidelines, state that a successful management plan of asthma should include symptom controls to minimise future risk of asthma-related mortality, exacerbations, persistent airflow limitation and side-effects of treatment [6]. The plan should include self-management education and skills training for patients and families/caregivers, effective medications, minimising existing and/or potentially modifiable risk factors and utilising non-pharmacological therapies and strategies. The British Thoracic Society/Scottish Intercollegiate Guidelines Network (BTS/SIGN) 2019 guidelines provide a diagnostic summary of clinical assessment and objective testing for asthma, including the positive test thresholds and algorithmic framework for objective tests for adults, young people and children (aged 5 and over) [7].

In the UK, children and adults of South Asian origin living with asthma are reported to experience worse outcomes in a number of domains. These differences are evidenced at the outset of a patient’s asthma journey – that of getting a diagnosis. Qualitative studies have reported concerns about delayed diagnoses amongst South Asians relative to other populations [8, 9]. Other studies report that, once a diagnosis is established, South Asians experience worse morbidity and mortality, mainly due to differing severity of asthma between groups with covariates including differences in health-seeking behaviour (e.g. self-management), difficulties in accessing high-quality primary care services, and variation of confidence in General Practitioners that may arise from the unfamiliarity of diagnosing and managing asthma in the SA population [10–12]. A meta-analysis found that South Asians had an odds ratio of 2.9 (95% confidence interval 2.4–3.4) of admission to hospital for asthma relative to white children and adults [13]. Despite this, South Asian children with asthma in England and Scotland were less likely to receive prescribed bronchodilators or anti-inflammatory drugs including steroids and antibiotics than a representative sample [14]. A further study of UK South Asian 2–4 year-olds found that they had an increased incidence of multiple wheeze but lower prescriptions of inhaled steroids compared to white children, likewise suggesting under-treatment [15]. It is known that children and carers of children with asthma already face difficulties due to the burden of long-term care and managing acute exacerbations, and this burden is exacerbated for children from South Asian backgrounds [13, 16]. Effective interventions to tackle these health disparities are urgently needed. To develop an effective intervention, a nuanced understanding of the patient and family/caregiver experience is key, alongside a thorough assessment of barriers and facilitators.

In any country, regardless of ethnicity, an individual’s culture, beliefs and attitudes, alongside those of their community, influence their health behaviour and engagement with the health care system. Simultaneously, it is evident that minority ethnic groups are often subject to a range of structural barriers to good quality healthcare [17–20]. The socio-ecological model suggests that health status and health behaviour are diversely influenced, from genetics to familial and community upbringing, psychology, biology, the environment, and the political and social context [21]. Through appreciating the broad array of factors that can influence an individual’s health, intervention planners can develop interventions tackling a problem at multiple levels, arguably more comprehensively and sustainably [22]. Yet, despite the importance of connecting with minority communities to understand these influences and develop services, they are under-represented in several areas of research, notably clinical trials [23, 24]. Nevertheless, multiple authors have described methods for involving minority communities in research and intervention development [25–27].

This approach is increasingly advanced as an essential part of national policy frameworks. In the UK, the Children and Young People’s Health Outcomes Forum report [28] and Healthy Lives, Healthy People [29] have made recommendations encouraging inter-service coordination across the health, social care, education and voluntary sectors, and stated that children and their families/caregivers should be involved with decision making, and given the opportunity to contribute to service improvements. These recommendations come alongside those of the UK Public Health Outcomes Framework [30], which states as a high-level outcome that there should be reduced differences in life expectancy and healthy life expectancy between different communities.

Notwithstanding growing evidence that culturally targeted interventions may increase engagement, this approach had been under-used by health promotion programmes, especially for tailoring that stretches beyond the individual to the community [31]. Additionally, few asthma studies have made use of participatory approaches to design with minority groups [32, 33]. This is despite qualitative studies reporting that participatory design can improve relations and involvement from hard-to-reach groups [34, 35]. Community-based participatory research (CBPR), one form of participatory research, can be particularly effective in achieving a successful culturally targeted interventions as it views research as a collective enterprise between researchers and the researched, taking place in familiar community settings [36]. Thus asthma, a long-term condition where existing interventions are or have infrequently been tested or tailored towards the substantial South Asian population in the UK, may greatly benefit from employing CBPR principles [37].

In recent years, several research groups have implemented culturally tailored interventions, with or without participatory design, A systematic review of culture-specific programmes for adults and children with asthma [38] found four relevant randomised controlled trials (RCTs) in 2009, and an additional three RCTs in a 2017 update (a total of seven), of which three involved South Asians (two in high-income countries) [39–41]. All seven RCTs provided individual and/or group-setting educational classes and support groups or materials for asthma management, while the control groups received either no intervention or partial intervention (e.g. standard education package rather than culturally tailored version) compared to intervention groups. Moreover, while the three RCTs that involved South Asians population were not participatory in design, the relevant studies included culturally tailored components via videos, graphically adapted materials and provision of bilingual trainers of asthma education. Additionally, several recent studies have been published utilising participatory approaches to intervention design for asthma in the USA; one in the Hispanic community, one in a predominantly African American population, and two in young people with no ethnicity specified [42–45]. However, we are not aware of a formal approach to participatory design in asthma in the South Asian population. Formalised collaborations between patients, their families/caregivers, clinicians, commissioners and policymakers make success more likely [46].

Intervention Mapping (IM) is another recently introduced developmental tool that addresses and assists in overcoming the challenges of combining evidence, theory and community-based participation during health promotion programme planning. IM charts the path from problem recognition to the identification of a solution and enables researchers to design interventions systematically - encouraging decisions to be evidence-based and theoretically informed. It is a structured process which provides protocols for making use of existing evidence and theory for the development of interventions [47]. It is also iterative, permitting movement back and forth between tasks and steps to make ongoing modifications throughout the development process. It can assess the current needs of a population, identify behaviours which may be detrimental to health, and what external factors influence these behaviours. It draws on health psychology theory in order to optimise the acceptability of a programme, and acknowledges the numerous social actors involved in healthcare delivery. Respect for such contexts is imperative amongst South Asian populations. IM has been adopted when developing interventions for mental health, nutrition, physical activity, and sexual health programmes [48–51]. IM has also recently been applied in a small number of cases in the UK [50, 52]. The Management and Interventions for Asthma (MIA) project was therefore devised, making use of CBPR principles combined with a modified IM approach to design an intervention to improve asthma management in British South Asian children.

## Methods

### Aims

The aim of the MIA project was to combine community-based participatory research (CBPR) principles with a modified IM approach, in order to develop an intervention planning framework for South Asian children with asthma. To ensure CBPR principles were observed, the MIA project involved all families in every stage of the research. South Asian children with asthma were chosen as the target population because of the existing evidence regarding inequities in outcomes in this group [53]. The study aimed to demonstrate how a tailored, collaborative approach could be used to design an acceptable intervention for a minority group.

### Design

The design of the MIA project followed Implementation Mapping processes. As mentioned, IM is a tool used for developing health promotion programmes in an iterative, structured manner across several steps from problem identification, to targeted intervention design [47]. The IM approach taken was modified; IM traditionally involves six steps, but since the MIA project aimed to assess methods used to develop a programme, the methodology was modified to focus iteratively on the first four steps, which focus on programme development.

In accordance with the socio-ecological model of health, the MIA project intended to formalise collaboration between all those involved in the care of the target children, including the children (patients) themselves, their families or carers, healthcare professionals (HCPs), and the wider community [21].

**Fig. 1:**
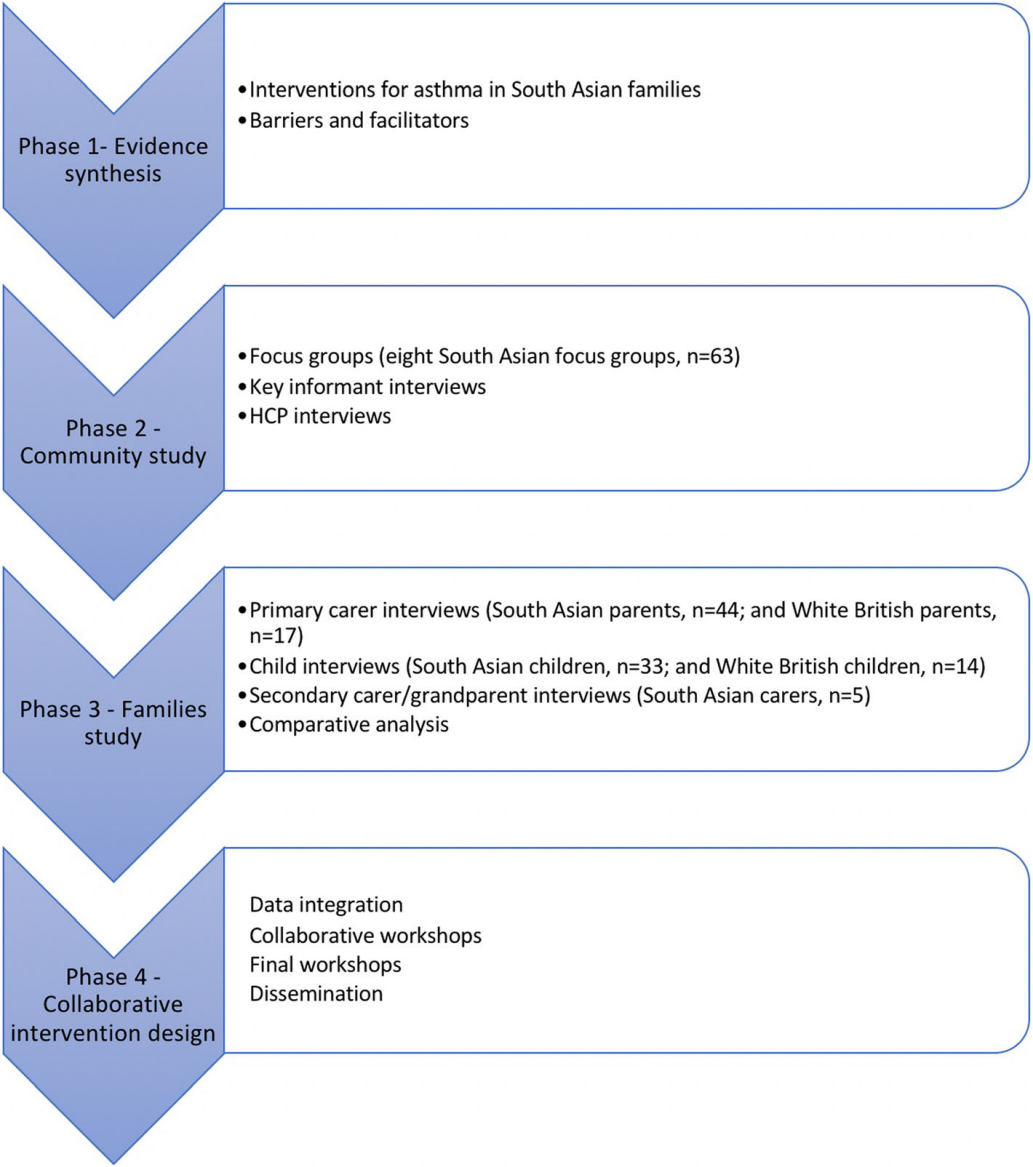
Stages of the MIA report: Phase 1, 2 and 3 inform phase 4

**Fig. 2:**
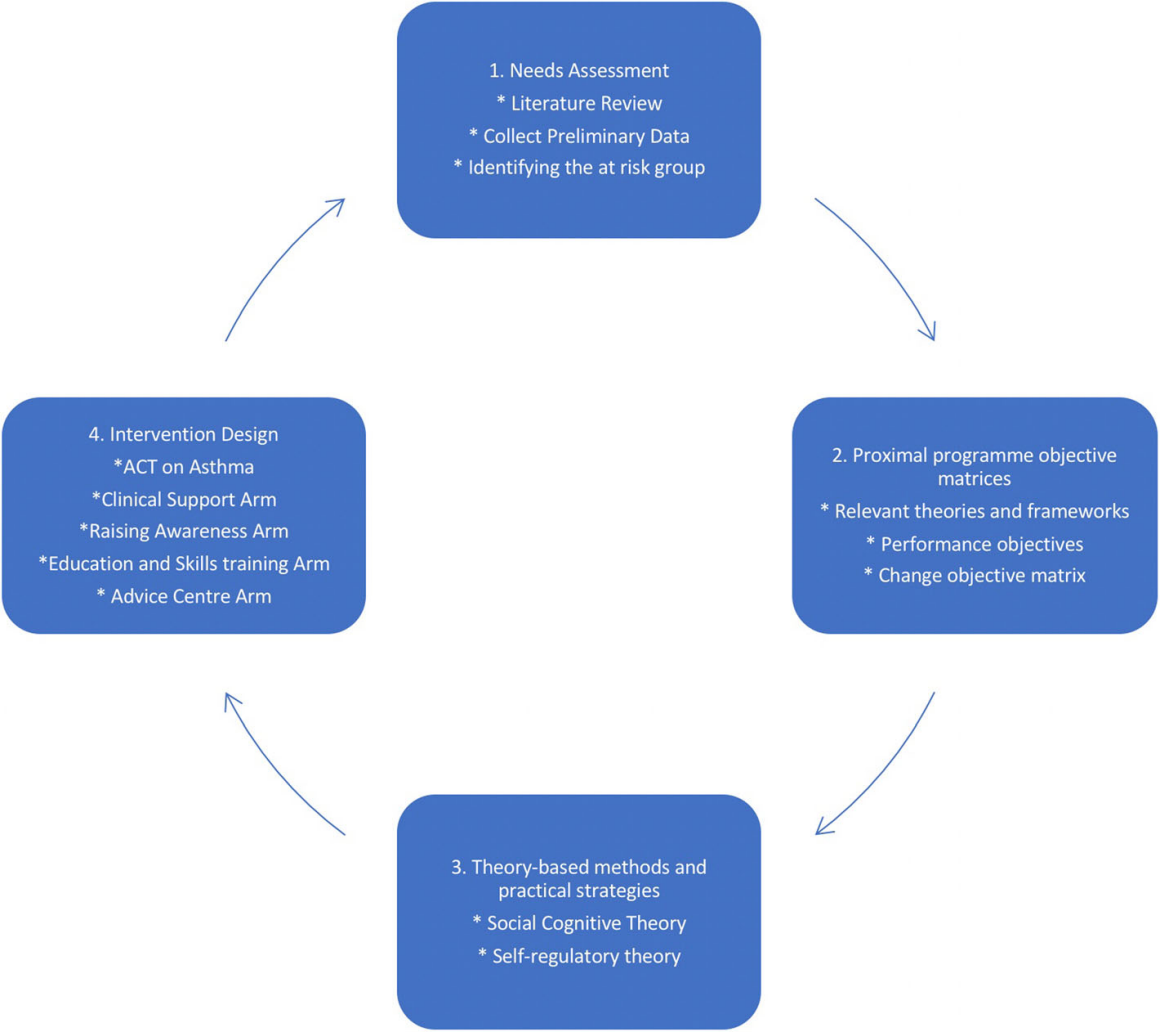
Modified Intervention Mapping process

The project was designed such that data gathered at each stage was used to guide the design of subsequent stages. Initially, a series of one-to-one interviews and focus groups were conducted supported by community facilitators, followed by workshops where the formative data was then presented back to the community, and health professionals. All community facilitators were bilingual members of South Asian communities who were trained as lay researchers to assist with all stages of the research. In addition, youth facilitators were given guidance on how to engage with the children and how to carry out the activities, with the research team supervising the youth facilitators. Written instructions for the activities were also supplied to the youth facilitators. The full details of each step are described in the following sections. An overview of the modified IM structure we followed is articulated in Fig. 1, and an overview of the process of data gathering in terms of interviews, workshops and analysis as applied to our study is described in Fig. 2. The results section of this paper describes in detail the process of implementation of this methodological approach.

### Setting and participants

This study was undertaken in a community setting within an urban environment in Leicester, UK. Leicester is a diverse city, with 45.1% of residents identifying as White British and 35.8% identifying as South Asian in the 2011 census, with the greatest proportion within this group identifying as Asian or Asian British: Indian (28.3%) [54]. The 35.8% also includes those of Bangladeshi, Pakistani and other Asian descent. Interview locations were primarily in community-run ‘South Asian’ centres, with some interviews taking place in participants’ homes. During the sampling protocol planning, the MIA team agreed to aim to interview up to a maximum of 30 primary carers and 30 children, and up to 10 secondary carers/grandparents (in order to gain perspective of family members other than the primary carer), unless data saturation was reached earlier.

Participants in the community study comprised 63 South Asian community members and 12 key informants; in-depth semi-structured interviews involved 30 South Asian families, 14 White British families and 37 HCPs treating South Asian children living with asthma; prioritisation workshops involved 145 SA, 6 White British and 37 HCP participants; 30 participants in finalisation workshops including 15 parents, 12 children with asthma, two HCPs, and one key informant. Several community-based organisations were actively involved in the recruitment or provision of key informants. These included a Temple (where study participants and key informants were recruited from); Muslim organisations (aided with recruitment and provided several key informants); and a Mosque (provided a key informant). A number of other local community centres were used for events and workshops as well as providing key informants.

Recruitment utilised a mixture of snowballing and purposive sampling, and varied by group. Snowballing permitted existing participants to recruit people they know to be in the study, and those new participants then to recruit people they know to be in the study, and so forth [55]. Focus group participants were recruited via snowballing, and also in response to posters, and through community facilitators. Family interviewees were recruited through snowballing, letters or direct approaches via GP/clinic or the research team, and community facilitators. The research team and community facilitators recruited key informants directly. HCPs were recruited through invitation letters, direct approach and snowballing. A number of participants who originally consented to do so did not ultimately participate; in the community study, 12 out of 75 participants did not attend the focus groups, 8 out of 38 recruited South Asian families were not interviewed. Some participants may have been known to the research team in a professional context prior to study commencement, e.g. through clinical encounters or as colleagues in the case of the HCPs. All participants involved in the early stages were invited again to participate in later phases, regardless of whether they were recruited before Phase 2 or Phase 3.

Attendance at interviews or focus groups were limited to interviewers, participants and, at times, community facilitators or other family members. The majority of interviews and focus groups were conducted/led by DB, who at the time was a Specialist Registrar in Paediatrics and Clinical Research Fellow. Participants were aware of the aims of the project and hence the aims of the research team, whose motivation for the project came through professional exposure and knowledge of existing evidence. A range of methods were used to ensure rigour in data collection and analysis processes. These included: the use of trained peer researchers; a collaborative design; end-user involvement; use of multiple data coders; digital recording of interviews and focus groups; consistency in researcher approach; use of NVivo to analyse data; coding and interpretation cross-checking (for a full overview, see [56]). The *COREQ* checklist for this paper is available as Additional file 1. Further details regarding eligibility and recruitment methods are available in previously published works [9, 56, 57].

### Data collection

At Phase 2, key informant interviews and community focus groups were recorded, with the direct transcription of English interviews and subsequent translation and transcription of interviews in other languages. The community focus groups discussions took place in both English and the appropriate language (Urdu or Guajarati), and for interviews with families and key informants, interviewees were offered their choice of language (English, Urdu and Gujarati). The interviews or focus groups undertaken in Urdu or Gujarati were translated into English by the community facilitators, and subsequently transcribed, and checked by bilingual members of the steering group and research team. The MIA team applied a thorough team cross-checking (with community facilitators and community representatives present) to receive feedback on the quality of the coding process, where a discussion was conducted until a consensus was reached in cases of ambiguity. For example, the research team had several meetings to discuss key issues emerging. The team then agreed on the list of issues for ranking, for example, from our analysis. At Phase 3, interviews were digitally recorded and transcribed; the resultant data were coded by one coder except when analysing differences between subgroups, where a second independent coder was used (e.g. South Asian vs White British). At Phase 4, data sheets were used, allowing participants to engage in ranking exercises. Interview questions not previously published elsewhere are available in Additional file 2*.* Interview questions and topic guides for children interviewed in Phase 3, and for the prioritisation workshops, are available elsewhere [57, 58].

The duration of interviews and workshops varied especially in the case of children, where the interview was conducted at the pace of the child. The thematic analysis took place using NVivo and the analytic process for the South Asian dataset opted an open coding process using the resultant framework, followed by the development of emergent themes and the clustering of themes in an interpretive process ([59]; see [56] for further description of processes observed). Participants did not review transcripts. To avoid undue influence from the existing South Asian interview coding, the White British interviews followed an independent process of open coding by a second analyst. The basic codes were then elaborated into a framework of thematic categories within NVivo; during this process, a small number of new nodes were added, for example, smoking during pregnancy and out-of-date medicine, although some nodes were applicable to South Asian data only (e.g. taking a child abroad). Subsequently, the generic codes that were applicable to both populations (e.g. decisions about taking medication) were inspected by both analysts to ensure consistency of meaning and validity of the thematic analytic process for both South Asian and White British datasets.

### Patient and public involvement

Developing the project together with community members to identify key issues is a powerful tool to enhance participation and enthusiasm and mobilise the community to improve an intervention’s effectiveness [25]. The original idea for the MIA project arose due to parental and community concerns about asthma management in SA population, alongside conversations with clinical leads. Therefore, the principles of CBPR (community-led identification of the issue in question) were applied. We were privileged to share the development process with the parent representatives and community facilitators (research partners of MIA). Two South Asian parents of children with asthma were involved at all stages of the research. One parent in the advisory group also participated in study recruitment, development of data collection tools and in the running of the children’s workshops. The parent representatives and community facilitators were integral to key stages of research, including identification of research questions, strategies to optimise recruitment, development of the intervention, interpreting and disseminating findings. They were trained by professional researchers in research methods and specialist knowledge of asthma and hence were a key part of the research team throughout the project. We viewed the partnership approach as essential in minimising the traditional power imbalance between researchers and the community being researched [60].

## Results

The phases of the project were as follows: 1) Systematic Evidence Synthesis, 2) Community study, 3) Families and healthcare professionals’ study, 4) Development of potential collaborative intervention strategies. These four phases describe the process of methodological tailoring and adjustment utilised to produce the final intervention.

### Phase 1 – systematic evidence synthesis

Phase 1 of the IM process facilitated the formation of collaborative partnerships with community members, key informants and stakeholders. We conducted a systematic review and evidence synthesis of barriers and facilitators to the management of asthma in South Asian children [61]. This review identified that no study had been published exploring knowledge of asthma amongst South Asian families living in the UK, although inadequate knowledge was evident in other countries. The study identified, in particular, barriers to asthma management, including lack of knowledge, communication difficulties, and non-adherence to treatment – there was less evidence regarding factors that facilitated successful asthma management in this group. These findings, alongside contributions from the project advisory group, were used to develop topic guides for a focus group study and schedules for individual interviews with key informants, healthcare professionals, parents/carers and children [62]. As part of this process, community members’ roles were developed, and the project aims were introduced.

### Phase 2 – community study

In accordance with IM practices, our needs assessment incorporated multiple perspectives (community knowledge, key informants’ knowledge, attitudes and perceptions, children’s experiences, healthcare professionals’ knowledge and experiences, and existing research) to gather evidence on the health problem in question, and to identify potential barriers and facilitators using a multilevel ecological approach.

The community study consisted of 8 focus groups with 63 members of local South Asian communities and one to one semi-structured interviews with 12 key informants considered to be knowledgeable about local communities, such as community centre managers. This constituted the first step in engaging communities and was intended to provide context for the work with families with a child with asthma. The community study aimed to understand perceptions of asthma amongst non-health professionals, as well as to consider community-level influences such as culture, education, religion, socio-economic status on the behaviour of children with asthma, and their families. Purposive sampling was used to ensure representatives were present from the different key South Asian ethnic, linguistic and religious groups, because experiences may differ across groups. Participants were not required to have personal or familial experience of asthma but were able to provide the community perspective towards asthma, and reflected on experiences of interacting with healthcare services from a South Asian perspective (see Table 1 and [56]).

**Table 1:** Demographics of the community focus groups used in phase 2 of the MIA project

Focus group	Ethnicity	Gender		Religions	Age range			
		Female	Male		18–34 years	35–54 years	≥ 55 years	Did not answer
1	Indian Punjabi	2	4	Sikh, *n* = 6	0	3	3	0
2	Indian Punjabi	6	4	Sikh, *n* = 4	5	2	2	1
				Hindu, *n* = 5				
				Did not answer, *n* = 1				
3	Indian Gujarati	8	0	Hindu, *n* = 7	1	4	3	0
				Muslim, *n* = 1				
4	Indian Gujarati	0	5	Hindu, *n* = 5	0	4	1	0
5	Pakistani (female group)	6	0	Muslim, *n* = 6	1	2	1	2
6	Pakistani (male group)	0	9	Muslim, *n* = 9	1	4	2	2
7	Bangladeshi (male group)	0	8	Muslim, *n* = 8	2	6	0	0
8	Bangladeshi (female group)	11	0	Muslim, *n* = 11	4	4	2	1
Totals	Eight groups	63 participants			14	29	14	6

### Phase 3: families and healthcare professional study

In-depth, semi-structured qualitative interviews with South Asian families and health care providers were carried out in Phase 3. The aims were to identify perceptions of asthma and to explore experiences of asthma amongst children with asthma and their families, as well as to understand perceptions and experiences of asthma and its management amongst HCPs caring for children with asthma. White British families were also interviewed as a comparison. Data were elicited from 30 South Asian families, including parents/carers and secondary carers and 33 South Asian children who were aged between 5 and 12 years (see Table 2), and 14 White British families and 14 White British children who were aged between 5 and 11 years old [9, 57]. In terms of asthma treatment, seven South Asian children were at British Thoracic Society (BTS) Level 1, 17 at Level 2, six at Level 3, three at Level 4 and none at Level 5. Fourteen White British children comprised three who were at BTS Level 1, eight at Level 2, three at Level 3, and none at Levels 4/5. BTS levels are stepwise management of asthma recommended by BTS/SIGN [7]. South Asian and White British children provided a proportionally good representation of asthma severity in the UK. Interviews were conducted with 37 healthcare professionals who had clinical or social responsibility for South Asian children with asthma (see Table 3). Validation was ensured by checking back with the clinicians that their messages had been accurately represented and heard.

**Table 2:** Demographic of South Asian and White British families

	South Asian	White British
Mothers	29	13
Fathers	15	4
Carers	5	–
Boys	20	8
Girls	13	6

**Table 3:** Number and type of Health professionals interviewed

Type of Health professional (n)	Number of (n)
GPs	5
Health visitor	1
Paediatric Consultants/Registrars	6
Foundation Year 1 doctor	1
Inclusion manager	1
Practice manager	1
Community/School Nurses	16
Community Pharmacists	3
Research assistant	1
Senior hospital play specialist	1
Clinical operational lead	1
**Total**	**37**

### Phase 4: development of potential collaborative intervention strategies (sections A-D)

#### Section A: key themes

Data were integrated from the previous phases by the research team to inductively elicit key factors that could be tackled to improve asthma management at the patient and their families/caregivers, provider, and health-care system levels. Data were integrated until no new themes were emerging from subsequent interviews or data collection, nor did they in fact emerge at the community workshops. Eleven key themes were identified, comprising: getting a diagnosis, understanding of asthma, appropriate information supply on asthma, day to day management of asthma, care quality, types of asthma services, the usability of asthma services, communication with nurses and doctors, asthma medicines, experiences at school, and community awareness.

#### Section B: prioritisation workshops

The key elicited themes were presented to participants at four workshops: three with laypersons (two for South Asian families, one for White British families) and one with healthcare professionals, to discuss key issues in managing childhood asthma and to prioritise and achieve consensus on which theme to prioritise when developing the subsequent intervention. One hundred and forty-five South Asian participants (from different cultural and religious communities) and 6 White British participants were involved in the prioritisation workshops.

Different prioritisation methods identified below were used for each group (adults, children, and healthcare professionals) to ensure that everyone was given a voice and all opinions were heard.

### Adult groups

Modified Nominal Group Technique (NGT) [63], enabling minority opinions to be heard, aimed to ensure that no opinions were dismissed. To further minimise this risk, discussion and revision elements of NGT used individual voting rather than table consensus. The methods used to ensure appropriate cooperation and prioritisation in the workshops are detailed in Table 4.

**Table 4:** Modified Nominal Group Technique

• Findings were presented orally with hard copies available at each table • Participants were grouped according to language requirements, with community facilitators providing translations where required • Ranking datasheets were used for a linear ranking of the key themes that were presented • Small group discussions followed and focussed on the need for an intervention to be developed for each key theme • Following the discussion, participants were asked to re-rank the themes, again using a linear ranking, and were given the opportunity to explain their reasoning • Data from the workshops were tabulated with a Borda approach [64] used to calculate overall priorities	

### Children’s groups

For children, methods which were mindful of age variation, understanding and ability were used [65]. We used discursive workshops and visual methods rather than didactic techniques. Children prioritised key themes via diamond ranking, known to be successful with children [65–67] since it allows equal positioning of some items, making ranking easier for child completion [68]. Given participants’ different ages and abilities, older children were able to choose between a linear ranking or a modified diamond ranking, and younger children used a modified diamond ranking. Children were also able to use a Budget Pie ranking technique, whereby each child could distribute a virtual wallet of £300 across different priorities [69]. The results of these ranking techniques were combined using the Borda approach [64].

### Healthcare professional groups

E-workshops were used for healthcare professionals to maximise participation, with a questionnaire link sent by email to all 37 participating healthcare professionals. Participants ranked the 11 themes using linear ranking, and were then given the opportunity to explain their reasoning. Once again, the Borda approach was used to combine ranking data [64].

Ultimately, the theme selected for further exploration, which was consistently ranked highly by families, was ‘getting a diagnosis’, and this theme was focused on for the subsequent intervention development phase (see Table 5). This diagnosis domain includes consideration of barriers to timely diagnosis and the need for accessible information about the diagnosis for families with asthma.

**Table 5:** Modified Nominal Group Technique

South Asian community rankings (***n*** = 62)	South Asian parents of children with asthma rankings (***n*** = 22)	White British parents of children with asthma rankings (***n*** = 2)
Getting a diagnosis	Getting a diagnosis	What to do day to day
Understanding what asthma is	Not all doctors and nurses treating asthma well enough	Getting a diagnosis
Types of services available for asthma	Types of services available for asthma	Medicines for asthma
Being able to talk to doctors and nurses	Being able to talk to doctors and nurses	Information and support for families
Not all doctors and nurses treating asthma well enough	Understanding what asthma is	School and my child’s asthma
Medicines for asthma	Medicines for asthma	Understanding what asthma is
Having suitable information on asthma	What to do day to day	Not all doctors and nurses treating asthma well enough
School and my child’s asthma	Having suitable information on asthma	Knowing about and using services for asthma
What to do day to day	School and my child’s asthma	Raising awareness and understanding about asthma
Community awareness of asthma	Being able to use the services	Knowing what to do in an emergency
Being able to use the services	Community awareness of asthma	

#### Section C: intervention development

The research team received specialist training from IM expert, Kay Bartholomew [47]. The proposed intervention was discussed in meetings of research team members, the research advisory panel, community facilitators and additional external advisors. The advisory panel represented the following professional areas: paediatrics, public health, general practice, commissioners and children’s services (including service managers, school nurses and paediatricians). South Asian parents of children with asthma were represented. During these meetings, a formulation-based approach was adopted [70]. The prioritised objective was matched, via psychosocial theories, to an evidence-based intervention [71]. During this phase, a scoping review was conducted to find previously tested interventions for asthma in children; a total of 408 interventions were identified, with four focusing on South Asian children. The scoping review considered how, and by whom, interventions were developed and where they were tested, and whether they were successful. At this stage, interview and focus group data from the previous phases were incorporated, to ensure that the intervention would be culturally acceptable.

In line with IM methodology [47], the theme of ‘getting a diagnosis’ was used in order to identify performance objectives and change objectives that would facilitate an improved diagnosis process for asthma. Members of the research team convened to consider what behaviours should be encouraged to achieve the overall desired outcome of the programme (performance objectives), by firstly considering the target group, their current behaviour, and what behaviours would be more helpful to achieve the overall desired outcome. Alongside this, determinants of behaviour were identified (e.g. lack of understanding of symptoms due to a communication barrier). They then considered the changes that should be made to achieve the overall desired outcome of the programme (change objectives). The needs assessment had highlighted that areas of focus should include knowledge and skills, self-efficacy, individual perceptions of risk, and expectations of the outcome. Therefore, the Self-Regulation Theory [72] and Social Cognitive Theory [73] were observed when creating the change objectives. Self-Regulation Theory constructs an individual as an active agent who responds cognitively and emotionally to health threats, in so doing appraising health status and utilising coping strategies to address them. Similarly, the Social Cognitive Theory appreciates that learning occurs in a social context with a dynamic and reciprocal interaction of the person, environment, and behaviour. The objectives and observations were then matched to practical applications (see Additional file 3 for a sample matrix).

Therefore, in line with the IM process and with consideration of the findings from the scoping review, the following components were prioritised as vital for the intervention – awareness, context, and training. In this setting, awareness refers to the appropriate knowledge to live with and manage asthma; context refers to structural and personal factors that may influence outcomes; and training refers to education as well as empowerment to self-manage the condition. The different components were designed to target multiple aspects of the socio-ecological model.

**Fig. 3:**
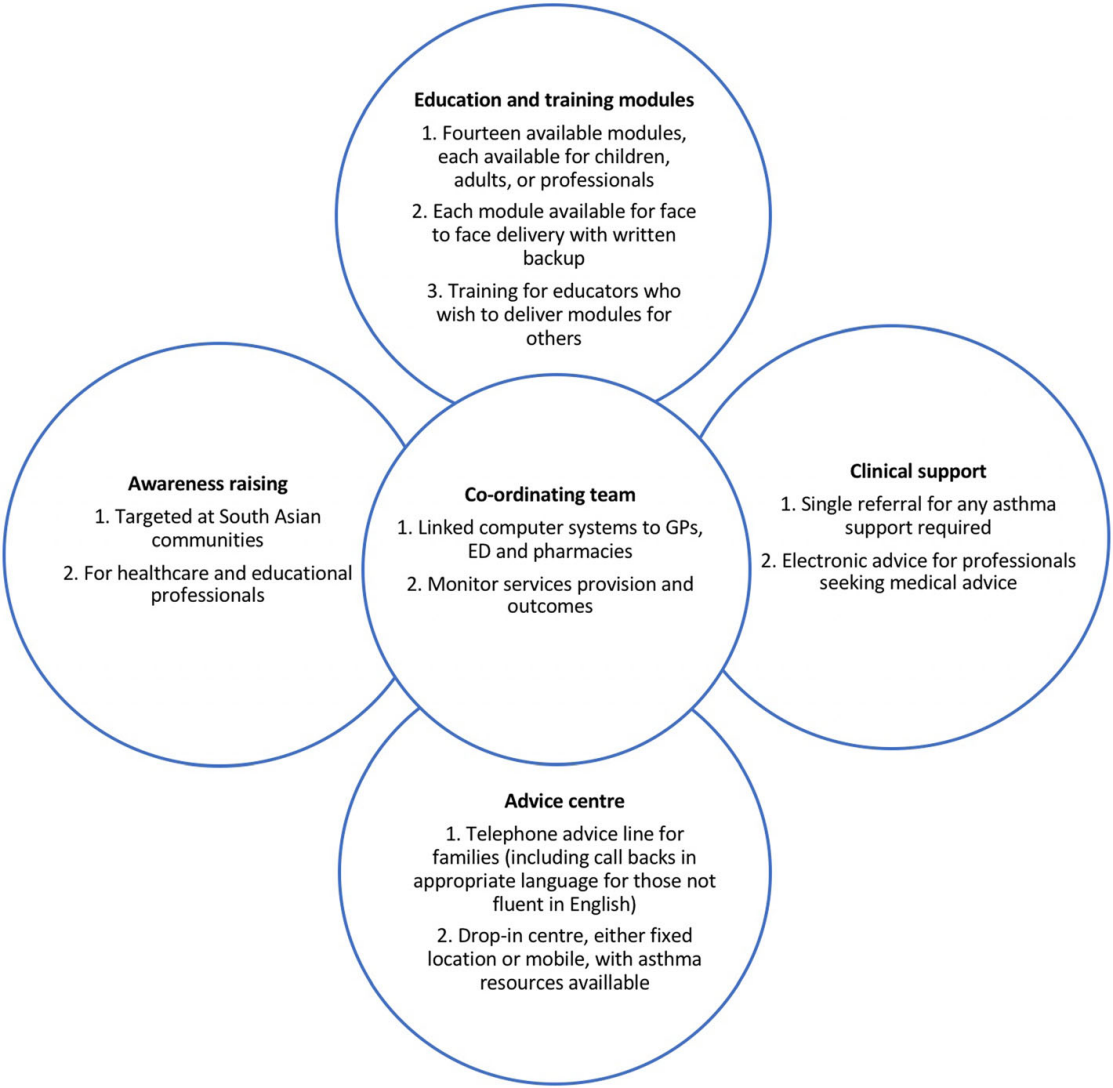
Finalised ‘ACT on Asthma’ programme

The intervention was therefore named the ‘ACT (Awareness, Context (cultural and organisational) and Training) on Asthma’ programme. ‘ACT on Asthma’ is designed to consider the wider factors that can facilitate a diagnosis of asthma, beyond simply medical investigations, including community awareness of asthma, education, accessibility and trust in medical services. The intervention was envisaged as a management pathway for asthma targeting each of these factors across three arms, led by a central co-ordinating team. The three arms at this stage comprised one arm targeted to raise awareness of asthma, both amongst communities and HCPs; one arm focusing on clinical support; and one arm focusing on education and training, again targeting both communities and HCPs (Fig. 3). The co-ordinating team was to provide the necessary oversight and integration required.

The elements of the intervention are designed to target both individual and systemic barriers that patients and HCPs may face, with training and education both of patients, their families/caregivers and of HCPs. The development of the programme adhered to participatory research principles, aiming to break down traditional power imbalances between researchers and the researched by using a bottom-up process of information gathering and intervention design, with consideration of socio-ecological factors.

#### Section D: intervention finalisation

The research team incorporated all the recommendations generated previously into the intervention plan. At this point, the intervention was carefully reviewed, discussed and refined. The draft intervention was taken for further refinement and finalisation to a combined professional and layperson workshop.

Thirty individuals took part in the finalisation workshops. These were 15 parents or carers of children with asthma, 12 children with asthma, two HCPs, and one key informant. In groups, they discussed one aspect of the intervention, their likelihood of using it, how they might improve it and any problems they predicted in its implementation. The finalisation process led to the addition of a fourth arm to the model, an ‘advice centre’ arm. The advice centre was envisaged as a hub which could act as a point of contact for patients and their families/caregivers when questions arose and could also host events where families could meet one another and share experiences (Fig. 3).

The modified IM approach was utilised to engage researchers, healthcare professionals and communities in the development of an intervention to address issues pertinent to asthma management for South Asian children and their families. The iterative process of intervention therefore produced a final four-armed ‘ACT on Asthma’ programme, with the four arms encompassing awareness, clinical support, training and education, and advice (Fig. 3). All arms would be co-ordinated by the central team.

The collaborative approach used involved multiple stakeholders in both the development and refinement of the intervention. Formative research methods were used to assess the needs of the population and the cultural context in which it must be delivered. We utilised two key principles throughout the development of the intervention; community-based participatory research principles and IM procedures. Using the community based participatory research approach, we were able to work closely with stakeholders and gain valuable sources of knowledge to inform the intervention development. This enabled us to engage, and accommodate key stakeholders; to incorporate the lived experiences of diverse groups of people; and to work towards the priorities of patients and their families/caregivers. In line with the project design, the process was systematic and informed by theory.

Findings revealed a consistent lack of lay understandings of asthma causes, triggers, symptoms, treatments and outcomes, which conflicted with medical definitions, suggesting a need for enhanced public awareness about asthma.

## Discussion

The aim of the MIA project was to develop a tailored intervention-planning framework, and then to use this framework to develop a multifaceted intervention programme. This paper illustrates how community-based participatory research principles were combined with the modified IM developmental tool to produce the ‘ACT on Asthma’ programme. ‘ACT on Asthma’ identifies, and targets, factors that prevent South Asian children with asthma from being managed appropriately. We hope the process and findings from this study will be useful for service providers and commissioners, and lead to the development and implementation of further evidence-based intervention programmes [56]. The Global Initiative for Asthma recognises that “*a person’s willingness and ability to engage in self-management may vary depending on factors such as ethnicity … interventions adapted for cultural and ethnicity perspectives have been associated with improved knowledge and significant improvements in inhaler technique*”, and that the patient-health care partnership should be tailored to individual patients’ cultural and educational backgrounds, beliefs and desire for autonomy. We believe the MIA project has contributed to the existing evidence regarding the burden of asthma for South Asian children; we note the current Global Initiative for Asthma Guidelines [6] do not elaborate or provide a specific reference in the text to South Asians. This study process provides information on the barriers and challenges they face and information on how to collaborate effectively with the SA population in regard to asthma management, and the practical process of co-developing interventions for asthma that are culturally acceptable.

The project was able to encompass the perspectives of communities, families, key opinion-formers, and health care professionals to attempt to ensure a rounded and comprehensive consideration of barriers and facilitators to asthma management and the development of proposed interventions. This ensured that a bottom-up approach to intervention development, placing the concerns and issues of families at its centre, and was also able to include professional and organisational perspectives. The term ‘South Asian’ refers to a heterogeneous group, and so this study aimed to include individuals across the main communities within this grouping, namely Indian, Bangladeshi and Pakistani. There were a high number of participants from ‘seldom heard’ groups, across several languages and religious/cultural backgrounds. The health priorities of South Asian community members have previously been demonstrated to differ from the priorities of HCPs, so incorporation of diverse views is highly important [58]. The use of community facilitators was also vital to conduct a culturally sensitive research project. As such, they were crucial members facilitating collaboration between PPI participants and the professional researchers, providing assistance with recruitment and acting as cultural mediators with the research team [74]. We acknowledge that the SA population is ‘heterogenous’ in origin and diverse in cultural specifics, but in the UK setting, there remains a degree of commonality of interest and experience, distinct from the ‘majority’ White British population in the UK, which serves to justify this conjoint approach within the group ‘South Asians’ [75]. We aimed for our intervention to be more culturally acceptable than current interventions/programmes which are designed and implemented with greater input from White British patients than from minority groups.

Practical challenges were tackled by using venues which served as hubs for the South Asian community, and by interviewing in participants’ homes and languages where necessary. Community venues were easily reached via public transport, and participants with particular needs were ordered taxis. Workshops were held at a time suitable to the participants, most frequently weekends or evenings, with financial recompense for transport costs. To help facilitate interviews with families, on-site crèche facilities were provided, alongside refreshments accommodating diverse dietary requirements according to religious and cultural beliefs. Moreover, low-value vouchers for local shops were provided for workshop participants and youth facilitators to thank them for their participation, and an on-site crèche was for provided for those who chose to use the facility.

A mixed-methods study evaluating relationships between community members and researchers in a community-based participatory research project reported that an important promoter of success occurs when partners feel strong motivation towards each other and the health topic in question [76]. The partners in the MIA project were particularly dedicated because of knowledge of the disparity in outcomes amongst this patient group. However, in the same study, self-rated perceptions of success also fell over time as problems were encountered [76]. This highlights the importance of maintaining energy and communication throughout a project. The study in question aimed to foster more formal academic relationships between researchers and the community through teaching in academic methods while preserving their non-academic knowledge that is highly valuable for the research team. In this study, this occurred with mutual learning through conversation between parent representatives and community facilitators and researchers, and if expanded could be a route that could encourage long-term sustainable relationships and be useful for programme implementation. Developing an equal partnership is particularly beneficial for the co-creation of knowledge that brings about lasting local change.

Going forward, implementation science research has a key role in developing health disparity research, or areas where outcomes for minority groups, as for South Asians in the UK with asthma, are worse than for majority groups [13]. For example, Chinman et al. have argued that most disparity research currently focuses on detecting disparities, rather than exploring the underlying mechanisms that may explain them or developing interventions to combat them [77]. They argue that, for example, frameworks such as the Consolidated Framework for Intervention Research may be useful in developing the focus of studies of the mechanisms of disparities because they focus on systemic factors beyond the patient, their families/caregivers and provider. A new framework proposed by Nápoles and Stewart describes a ‘transcreational’ framework to design and deliver interventions to reduce disparities within communities, which engages community partners from the beginning of the programme [78].

A major strength of IM as a tool for intervention design is that it provides both a theory and evidence-based planning process to develop tailored interventions with potential cost-offset. A systematic review has reported on significant increases in uptake of disease-prevention interventions designed using IM [79]. However, a particular challenge when using participatory research methods, coupled with IM, is that project scope can grow, particularly when working with a previously under-researched population and when certain needs are not anticipated a priori. Also, although this study identified a number of areas of focus for South Asian children with asthma, it would have been unfeasible to tackle all areas at once, hence the selection of the ‘getting a diagnosis’ exemplar. IM is resource intensive as it requires lengthy analysis as well as maintenance of a high degree of community involvement over the project’s lifetime. For instances, the planned study timeline was about 2 years and 5 months in total (see Additional File 4). Notably, we utilised a great deal of resources that ultimately could only be focused on one area for the South Asian population. However, a benefit of conducting this type of study was that we were fully able to appreciate the real-world feasibility of conducting intervention development in this manner. Organising a series of workshops to develop and finalise the programme was also costly in terms of hosting and reimbursing participants. It can be questioned whether the ultimate outputs are cost-effective [80]. These challenges, in conjunction with growing evidence on the benefits of tailored interventions, need to be fully appreciated when planning an IM approach. Further consideration should be given to how such a formulation-driven approach can be both rigorous in understanding barriers and levers to change, yet less resource intensive (e.g. projects with extensive health economic modelling).

There are, therefore, a number of limitations to this study. The MIA project highlights how taking a collaborative, multifaceted approach to intervention design can result in the development of a comprehensive intervention, but further research is necessary to establish whether such efforts are justified in terms of efficacy and cost-effectiveness. The MIA project was not powered or funded to test a clinical intervention, rather we aimed to offer a methodology in Asthma diagnosis and management based on a selected single issue, in the hope that it could provide a framework for intervention development in this population later on, either with the selected issue we used, or another issue. The intervention produced through this process has not yet been implemented in practice. Additionally, although we aimed to incorporate patients and families/caregivers from seldom-heard backgrounds, our sampling may not have been representative because those who are likely to engage in such research projects may have characteristics that differ from those who do not. Unlike randomised-controlled trials, selection bias is not necessarily a marker of unreliability in a participatory study; however, some limitations may have been introduced since the approach for recruitment in Phases 3 and 4 included inviting back participants from Phase 2 (participants who experienced interviews or focus group sessions positively may be more likely to accept subsequent invitations) and some participants were known to the research team in a clinical context prior to study commencement. The resultant data was coded by one coder from each population dataset (one for SA participants, one for White British), although, this was minimised via subsequent revisits by both analysts to ensure that integrity of the coding was maintained and consistent. Finally, we do not have information on the number of participants approached who declined to participate, and do not have further information on the demographics of participants beyond what is presented.

Authors felt that it was essential that our findings be disseminated back to the target population. Dissemination included local dissemination to participants through our workshops, dissemination meetings with academics, UK and international academic conferences, wider dissemination in media (e.g. BBC and independent radio, Asthma UK grant application), and use of findings for further research applications [56]. Regarding the implementing of interventions by the community, some participants helped to direct an awareness film, which was freely available for the community and other stakeholders through the internet. Asthma UK representatives attended a number of workshops, enabling the community to directly engage and receive further information about their child’s condition. Moreover, some of the community members were supported to raise awareness about asthma within their child’s school. Finally, as previously cited, the MIA project resulted in academic publications [9, 56–58, 61].

In terms of impact, a number of other studies have cited our methodological approach including studies examining paediatric autopsies within minority groups [81, 82], participation in research amongst children in life-limiting conditions [83], and a US-based study on an asthma toolkit [84]. The approach has been cited in local meetings in London boroughs with diverse populations, and the methodology is being used in upcoming UK and India-based studies of childhood infections, pollution and nutrition [85–87]. Further post-hoc analyses on this utility of this approach compared to others would be valuable, with acknowledgements of the difficulties of its application. The procedures used in the MIA project can be adapted using the principles described and applied in other settings with other South Asian and other culturally distinctive groups, with due attention to such local issues as the mix of ethnicity, language, religion and history.

## Conclusions

The strength of the MIA project comes from its rigorous engagement of South Asian children, families and communities affected by asthma, and its identification of their needs and concerns. The MIA project identified barriers in relation to individual knowledge and understanding of asthma management, inadequate education of health care professionals on the needs a minority community, and barriers relating to the way in which asthma services are provided. The subsequent intervention was developed with, and revised inductively alongside, patients, their families/caregivers, and the HCPs who manage this common condition. We recommend that this collaborative and participatory approach is considered by intervention developers and programme planners, especially when working with previously neglected patient groups, alongside rigorous and continuous evaluation of efficacy.

## Supplementary information

**Additional file 1.** COREQ Checklist. Consolidated criteria for reporting qualitative studies (COREQ): 32-item checklist. (PDF 113 kb)

**Additional file 2.** Interview Questions and Topic Guides. A document providing details of scripts and topic guides used in interviews and focus groups during the study. (DOCX 21 kb)

**Additional file 3.** Sample matrix of performance objectives, determinants and change objectives. Sample matrix of performance objectives, determinants and change objectives. (PDF 138 kb)

**Additional file 4.** Timeline for Study - Gantt Chart. Gantt chart. (PDF 94 kb)

## Data Availability

The datasets used and/or analysed during the current study are available from the corresponding author on reasonable request.

## Abbreviations

ACT: Awareness, Context (cultural and organisational) and Training
BBC: British Broadcasting Corporation
BTS: British Thoracic Society
CBPR: Community-Based Participatory Research
GPs: General Practitioners
HCPs: Healthcare Professionals
IM: Intervention Mapping
MIA: Management and Interventions for Asthma
NGT: Nominal Group Technique
RCTs: Randomised Controlled Trials
SA: South Asian
SIGN: Scottish Intercollegiate Guidelines Network
UK: United Kingdom
WB: White British
