# Supplementary material for: A structured collaborative approach to intervention design using a modified intervention mapping approach: a case study using the Management and Interventions for Asthma (MIA) project for South Asian children
Source: BMC Med Res Methodol. 2020 Nov 2;20:271. doi: 10.1186/s12874-020-01148-y (PMC7607819; doi:10.1186/s12874-020-01148-y)
Supplement: Supplementary file 1 — Additional file 1. COREQ Checklist. Consolidated criteria for reporting qualitative studies (COREQ): 32-item checklist. (PDF 113 kb) [file 12874_2020_1148_MOESM1_ESM.pdf]

Additional file 1: Consolidated criteria for reporting qualitative studies (COREQ): 32-item checklist

Developed from:
Tong A, Sainsbury P, Craig J. Consolidated criteria for reporting qualitative research (COREQ): a 32-item checklist for interviews and focus groups. *International Journal for Quality in Health Care*. 2007. Volume 19, Number 6: pp. 349 – 357

**No. Item**	**Guide questions/description**	**Reported on Page #**
**Domain 1: Research team and reflexivity**		
Personal Characteristics		
1. Interviewer/facilitator	Which author/s conducted the interview or focus group?	Line 172-174
2. Credentials	What were the researcher’s credentials? E.g. PhD, MD	Line 172-174
3. Occupation	What was their occupation at the time of the study?	Line 172-174
4. Gender	Was the researcher male or female?	Line 172-174
5. Experience and training	What experience or training did the researcher have?	Line 172-174
Relationship with participants		
6. Relationship established	Was a relationship established prior to study commencement?	Line 162-163
7. Participant knowledge of the interviewer	What did the participants know about the researcher? e.g. personal goals, reasons for doing the research	Line 171-176
8. Interviewer characteristics	What characteristics were reported about the interviewer/facilitator? e.g. Bias, assumptions, reasons and interests in the research topic	Line 171-176
**Domain 2: study design**		
Theoretical framework		
9. Methodological orientation and Theory	What methodological orientation was stated to underpin the study? e.g. grounded theory, discourse analysis, ethnography, phenomenology, content analysis	Line 141-149
Participant selection		
10. Sampling	How were participants selected? e.g. purposive, convenience, consecutive, snowball	Line 157
11. Method of approach	How were participants approached? e.g. face-to-face, telephone, mail, email	Line 157-164
12. Sample size	How many participants were in the study?	Line 248-250
13. Non-participation	How many people refused to participate or dropped out? Reasons?	Line 164-166

Setting		
14. Setting of data collection	Where was the data collected? e.g. home, clinic, workplace	Line 139, 143-144, 155-156
15. Presence of non-participants	Was anyone else present besides the participants and researchers?	Line 171-172
16. Description of sample	What are the important characteristics of the sample? e.g. demographic data, date	Tables 1-3
Data collection		
17. Interview guide	Were questions, prompts, guides provided by the authors? Was it pilot tested?	Additional file 2
18. Repeat interviews	Were repeat interviews carried out? If yes, how many?	N/A
19. Audio/visual recording	Did the research use audio or visual recording to collect the data?	Line 179-191, 190-192
20. Field notes	Were field notes made during and/or after the interview or focus group?	Line 179-191, 190-192
21. Duration	What was the duration of the interviews or focus group?	Line 196-197
22. Data saturation	Was data saturation discussed?	Line 144-147
23. Transcripts returned	Were transcripts returned to participants for comment and/or correction?	Line 200
**Domain 3: analysis and findings**		
Data analysis		
24. Number of data coders	How many data coders coded the data?	Line 190-192, 511-513
25. Description of the coding tree	Did authors provide a description of the coding tree?	N/A
26. Derivation of themes	Were themes identified in advance or derived from the data?	Line 279-287
27. Software	What software, if applicable, was used to manage the data?	Line 197
28. Participant checking	Did participants provide feedback on the findings?	Line 186-188
Reporting		
29. Quotations presented	Were participant quotations presented to illustrate the themes/findings? Was each quotation identified? e.g. participant number	N/A; manuscript is programme overview incorporating sub-studies
30. Data and findings consistent	Was there consistency between the data presented and the findings?	Results section; Line 317-319
31. Clarity of major themes	Were major themes clearly presented in the findings?	Line 317-319
32. Clarity of minor themes	Is there a description of diverse cases or discussion of minor themes?	Table 5
