# Supplementary material for: A structured collaborative approach to intervention design using a modified intervention mapping approach: a case study using the Management and Interventions for Asthma (MIA) project for South Asian children
Source: BMC Med Res Methodol. 2020 Nov 2;20:271. doi: 10.1186/s12874-020-01148-y (PMC7607819; doi:10.1186/s12874-020-01148-y)
Supplement: Supplementary file 2 — Additional file 2. Interview Questions and Topic Guides. A document providing details of scripts and topic guides used in interviews and focus groups during the study. (DOCX 21 kb) [file 12874_2020_1148_MOESM2_ESM.docx]

Additional File 2: Interview Questions and Topic Guides

Phase 2: Key informant interviews

1. Could you tell me a little bit about your role in the community and what you do?
i. Probe: How long have you been working in this or a similar role?
ii. What contact do you have with children/families?

2. In your experience, have you met or worked with any children with asthma or their families? Could you describe a typical child with asthma?

i. Probe: What are the key features of asthma?

ii. Do you have any sense of the numbers of children with asthma in the community?

3. How do you think parents in your community might feel about their child having asthma?

i. Probe: Might they be self-conscious/embarrassed/worried about their standing in the community?

4. What do you think that the average person in the community thinks about children with asthma?

i. Probe: Would anyone treat the child or their family differently because they have asthma?

ii. Do you think that people in the community understand what it’s like to have a child with asthma?

iii. Do you think that people in the community understand what children with asthma can and can’t do?

5. Do you think that someone’s cultural background, being . . . has any effect on the way they think about asthma?

i. Probe: What about what causes asthma?

ii. About treatments for asthma?

iii. About what’s normal/expectations for a child with asthma?

iv. In relation to how families are treated in the community?

6. Do you think that a person’s religion makes any difference to how they view asthma, or creates any problems?

7. Does being . . . have any impact on how the child is treated by others, including professionals?

8. Probe: Is there a perception of prejudice amongst doctors and nurses?

Research shows that sometimes parents find it very difficult to have a child with asthma.

1. What do you think might be some of the problems they face?

i. Probe: Within the family
ii. Within the community
iii. With professionals – at diagnosis, getting treatments

2. As far as you know, what kinds of treatments are there for asthma?
i. Probe: How are treatments viewed by the community?
ii. Problems with western medicines, especially steroids?

iii. What might stop someone from using their medicines?

iv. Use of alternative medicines?

A very important part of this research is identifying ways that we can help young children with asthma and their families.

1. What information or support, if any, should be offered to parents and children if diagnosed with asthma?

2. What do you think the NHS – doctors and nurses – could do differently that might help families in your community?

3. What do you think the community might be able to do that could help?

4. What could someone in your position do that might be useful?

5. What do you feel is the best way to involve people in the community in any of these suggestions? (Depending on answer . . .) Why would that work?

Phase 2: Community focus groups

Firstly, I’d like to welcome you all to the group and thank you for agreeing to speak to us. The reason we’re here is because a team of researchers from the University of Leicester, De Montfort University, and the NHS are trying to improve the care of Indian/Bangladeshi/Pakistani children with asthma. We would like to know what people think about children and families who have asthma, and to ask for your help in improving the lives and care for children with this problem. This is important because asthma affects lots of children and can be very difficult for children and parents.

Don’t worry if you think you don’t know anything about asthma – we have lots of different things to talk about and you might find that you know more than you think. We’ll also be using a story about a boy called Samir to discuss some of the different ideas. If you do know someone with asthma or have asthma yourself and would like to know more about the things we talk about today, we have some information leaflets that we can give out at the end of the session.

Before we begin I would like to remind you that whatever you say here will be kept confidential. I hope that you will feel free to express your views. Your names and personal details will not be mentioned in any report. I am using a tape recorder to record our conversation because it is difficult for me to write down everything you say. Please try to speak one at a time so that I can follow what is being said.

1. Let’s start with everyone telling us their name and whereabouts they were born? I’ll go first . . .

2. Can everyone think of a child that they know? Can you describe them briefly? I’ll go first . . . (say one or two sentences about a child you know e.g. I’m thinking of my niece, she’s 6 years old and very lively, never sits still and never stops talking).

3. If I were to say ‘this is a healthy child’, what would this mean to you?

4. You may have heard the word ‘wheezy’ (in translation or in English if no translation) used to describe a child. What does wheezy mean to you?

5. Many of you may have heard about a condition called asthma. In your own words, can you describe a child with asthma?
i. Probe: Is wheezy the same as asthma?
ii. Is asthma common?
iii. How do you know if a child has asthma?
iv. What kind of things can happen to someone who has asthma?

I’m going to tell you a bit about a boy called Samir. Samir is 6 years old. Samir’s always been prone to chesty coughs and often wakes up at night coughing. Samir often can’t keep up with his brothers and sisters when playing and gets out of breath very easily. Samir’s parents took him to their local GP, who said that Samir has asthma.

6. What do you think that Samir might think about having asthma?
i. Probe: Might he worry about anything?
ii. What about his friends?
iii. What about sports?
iv. Might he be thinking about school?
v. What about going to see doctors?

7. If you were Samir’s mother or father, how would you feel about having a child with asthma?
i. Probe: Might it affect the relationship between you and your husband/wife?
ii. Might it affect your relationships with other people in the community?
iii. Might it affect your standing in the community?
iv. Would you want to seek advice from anyone else?
v. Might it affect Samir’s future career prospects?
vi. Might it affect Samir’s future marriage prospects?
8. How might having one child with asthma affect the rest of the family?
i. Probe: Might it affect going out to a family function, like a wedding?
ii. Might it affect the relationships between Samir and his brothers and sisters?
iii. What about the extended family?
iv. Might the family change the way they treat Samir?

9. What do you think that an average person in the community might think about a child with asthma?
i. Probe: Does it matter?
ii. Would they be seen as healthy?
iii. Would they be seen as normal?
iv. Would anyone treat the child differently because they have asthma?
v. Would anyone treat the family differently?
vi. Is there a stigma attached to asthma?
vii. Would anyone worry about having that child visit their house?
viii. What about staying in their house?
ix. Would anyone worry about their child marrying someone with asthma?
x. Would anyone worry about hiring a young person with asthma?

10. Do you think that being Indian/Bangladeshi/Pakistani means that you think differently about asthma to someone who is not from this background?

11. Do you think that being Indian/Bangladeshi/Pakistani influences the way people respond to a child or family with asthma?
i. Probe: (If yes or no) – why is that?
ii. Does age make a difference to what people think?
iii. Does being born in the UK or in India/Bangladesh/Pakistan make a difference?
iv. Does religion make a difference?
v. Does being male or female make a difference?
vi. Does your job/role in the community make a difference?

12. Do you think that doctors might respond differently to a child who’s from an Indian/Bangladeshi/ Pakistani family, compared to a child who’s not from this background?
i. Probe: Does the doctor’s ethnicity make a difference?
ii. What about nurses?
iii. What about receptionists?

13. If parents want to get treatment for their child with asthma, how do they do this?
i. Probe: Where would most parents go to for treatment?
ii. Who would most parents go to for treatment? The GP gave Samir two inhalers to use; a brown one to take every day, and a blue one to take whenever he’s wheezy (hold up example inhalers).
14. What do you know about these medicines?
i. Probe: Do they work?
ii. Are there any problems with them?
iii. Are there any times or places that you can’t take them?
iv. Would it be better to take the medicine in a different way?
v. If yes, why?
15. Are there any concerns about a child with asthma taking medicines every day, even if they seem OK?
i. Probe: Is it better to only take medicines when they’re wheezing? Why?
ii. Does the type of medicine, e.g. steroid (show the brown inhaler again) make a difference? iii. Are there any alternatives to using medicines for asthma and wheezing?
16. Is there anything that the NHS could do to help children who have asthma?
i. Probe: What about making appointments with GPs?
ii. What about going to see specialist children’s doctors?
iii. What about going to see specialist asthma nurses?
iv. Is it important whether you see a doctor or a nurse?
v. Is it important whether you see a GP or general paediatrician, or an asthma specialist doctor?
vi. Do you think that where the clinics are held makes a difference?
17. Is there anything people could do to themselves?
i. Is there anything children could do for each other?
ii. If there anything families could do?
iii. Is there anything communities could do?
18. Is there any need for more information about asthma in children and families?
i. Probe: Do you think that people in the community understand what it’s like to have a child with asthma?
ii. Do you think that people in the community understand what children with asthma can and can’t do?
iii. What information is there already?
iv. What kinds of things do you think people need to know about?
v. Why are these things important?

19. What is the best way of getting information to families and communities?

20. Is there anything else that you feel it would be useful for us to know about a child with asthma in your community?

Summary

The intention tonight was to talk about how the Indian/Bangladeshi/Pakistani community views asthma. We’ve talked about the symptoms of asthma, about where and how to get treatment, and how having asthma affects a child and their family. We’ve also talked about how this community might see a child with asthma and what the community could do to help. Have we missed anything? Is there anything else you’d like to add before we finish? Thank you very much for your time. You will be given a voucher for your involvement in this focus group. If you would like any further information about asthma, please stay behind for a minute and I will provide some.

Phase 3: Family interviews (parents/carers)

Hello. Thank you very much for agreeing to speak to me. We’re going to talk about you and your son/ daughter (say name) and their breathing. I’d like to talk about when things first started, then we’ll move on to what things are like now. I’ll also ask some questions about what happens when he/she has a bad day, and about any treatments you use.

Please feel free to say whatever you’re thinking and please remember that everything you say will be kept confidential. If at any time you’d like to stop or take a break, just let me know and we’ll do just that.

Let’s start by talking a little bit about your family. How many children do you have? Who lives in your house? Does anyone else help you to look after your son/daughter?

1. When did (child’s name)’s breathing problems first start?
– Initial symptoms/recognition
– Who/how diagnosed
– Information/support provided
– Barriers to diagnosis: Seeking help; HCP attitude; Access to service

2. People have lots of different ideas about why children get wheezy. Why do you think children get wheezy?

– Triggers vs. causes

– Heritability

– Contagion

– Gods will

– Diet

3. What do you think causes (child’s name)’s asthma/wheezing?

4. Could you describe what (child’s name)’s asthma is like on a day-to-day basis?

– Symptoms

– Impact on day-to-day life: Lifestyle restrictions; Time off work; Family life; Schooling

5. Who makes the decisions about day-to-day things?

– Mum/dad

– Child

– Extended family

6. Could you describe a recent asthma attack? (substitute bad attack if little response)

– Describe management: Use of health service; Use of tests/diagnostics; Role of extended family in management

7. Problems faced

– Recognition of need to seek help

– Access to service A&E/walk in/GP

– Practicalities – finances, time of day, weekends, childcare

– HCP communication/attitudes/ethnicity/gender

8. What worked well?

– Good service

– Good person

9. Information provided

– What

– From who

– Useful/not

– What else wanted

– Timing

10. Solutions used/wanted

11. Let’s talk about treatments. What do you hope treatments will do for (child’s name)’s asthma/wheezing?

– Personal goals of treatment

– Management vs. cure

– Concept of asthma control

– What is good control?

12. Does (child name) take any medicines for his/her asthma? (substitute inhalers if no response) (go through medicines one by one)

– When do you use this/how do you feel about it/any problems getting child to take it/how do you remember to take it/does everyone in family know about it/use in public

– For preventer medicines: when do you stop/who makes decisions/side effects

– Specific attitudes: Longterm med use; Inhaler vs. tablets; Steroids

13. Who has helped you learn about the wheezing/asthma?

– Sources of knowledge

– Skills

– Overseas advice

– Role of schools/community centres/religious centres

– Use of written management plans

14. What do the rest of your family think about (child’s name)’s asthma?

– Who knows/gets involved?

– What does child think?

– Does anyone offer advice/do you follow it?

– Impact on management?

15. What about your friends?

– Do you tell others/stigma?

– Would you tell about another illness/specific to asthma

– Would it be the same for an adult illness? Future marriage?

– Common beliefs about causes

– Common attitudes towards asthma/children with asthma

– Advice offered/follow it?

– Has anyone upset you?

– Do cultural attitudes influence management?

16. Where do you go for help about (child’s name)’s asthma?

– Usual source of care

– GP/A&E/walk-in/family and friends/internet/religious leaders

– Who do you go to?

– What is the key issue – opening hours, location, relationship, gender, ethnicity

– Problems faced getting help: Communication with HCPs; Getting prescriptions

17. What would help overcome these?

18. Is there anything that’s you’ve found particularly helpful in taking care of (child’s name)’s asthma?

– What/why?

– Alternative therapies used: What/why; Diet; Heating/furnishing changes; Barriers or facilitators to using different options

19. Is there anything else we could do to help you cope with (child’s name)’s asthma?

– What/Why/How: Specific to your family; General suggestions for families and children; Specific points for Indian/Pakistani/Bangladeshi families

Phase 3: Healthcare professionals

Hello. Thank you very much for agreeing to speak to me.

The MIA research group are investigating the barriers and facilitators to asthma management in south Asian children with a view to developing an intervention programme that will be both practical and suitable. We’ll be discussing your experiences of managing asthma with south Asian children and families, in particular, any barriers you’ve faced and if, how or why these issues have affected your management. Towards the end I will ask about elements you’d like to see improved and how we might work together to achieve this.

1. Please tell me about your experience working with South Asian children and families with asthma

– Diagnosis: Use of testing; Acceptance; Discussion/information given

2. Communication

– Language

– Attitudes

3. Particular problems encountered

4. Particularly helpful aspects

5. How much do you feel you know about the lives of south Asian families?

– Confidence in knowledge

– Confidence in achieving working relationships

– Particular issues or helps around achieving working relationship with South Asian families

6. Is there anything you do differently for south Asian families?

– Modification to communication style, information given, choice of treatments

– Why?

7. What barriers do you think parents of south Asian children with asthma face in managing their children?

– Practical barriers

– SES/education/place of birth

– Cultural factors

8. Do you feel that there are any organisational barriers affecting how you manage asthma in south Asian children?

– Resources

– Time

– Training needs

– Interpreters

9. What would you like to see made better?
