# Supplementary material for: A structured collaborative approach to intervention design using a modified intervention mapping approach: a case study using the Management and Interventions for Asthma (MIA) project for South Asian children
Source: BMC Med Res Methodol. 2020 Nov 2;20:271. doi: 10.1186/s12874-020-01148-y (PMC7607819; doi:10.1186/s12874-020-01148-y)
Supplement: Supplementary file 3 — Additional file 3. Sample matrix of performance objectives, determinants and change objectives. Sample matrix of performance objectives, determinants and change objectives. (PDF 138 kb) [file 12874_2020_1148_MOESM3_ESM.pdf]

Additional file 3: Sample matrix of performance objectives, determinants and change objectives

| The actor | What do people/organisations do? | Why do people do that? (Determinants) Parental Perspective | Child's perspective | HCP perspective | Community perspective | Why does this matter? |
|---|---|---|---|---|---|---|
| **Parent** | Don't present to the GP with initial symptoms | Misattribution of symptoms: they think it's a cold or cough and therefore suitable for home management; don't think it is something to be concerned with | | Communication barrier: some words do not translate, not known in South Asian communities, parents don't speak English | Misattribution: mixed up between cough/cold/chest infection; give other causes such as damp house | Chronic symptoms are being seen as normal or being attributed to the UK climate/housing conditions. Not being recognised as potential treatable illness. Then exacerbated by overlap between colds/asthma attacks; families feel no value in attending GP as will be dismissed |

| The Actor | What do we want them to do instead? (Performance outcomes) Parents | Child | HCPs | Community |
|---|---|---|---|---|
| **Parents** | To recognise potential symptoms from normal behaviour | | Enable families to present repeatedly | Be aware of potential symptoms of asthma |

| The actor | (Change Objectives) Knowledge and Skills | Outcome Expectations | Self-efficacy | Risk perception/Fear | How should we do this? (Theoretical Model) Examples | Interventions or Theoretical model if no good example intervention |
|---|---|---|---|---|---|---|
| **Parents** | Parent: ability to identify symptoms of asthma | Parents: believe that going to GP has value to them and their child | Parents: believe that they are able to have an effective conversation with their GP | Parents: perceive that without treatment asthma could be dangerous to their child | Knowledge of asthma symptoms: modelling behaviour | Community vignettes |
