# Supplementary figures and images for: A structured collaborative approach to intervention design using a modified intervention mapping approach: a case study using the Management and Interventions for Asthma (MIA) project for South Asian children
Source: BMC Med Res Methodol. 2020 Nov 2;20:271. doi: 10.1186/s12874-020-01148-y (PMC7607819; doi:10.1186/s12874-020-01148-y)

Additional file 4. Timeline for Study - Gantt Chart

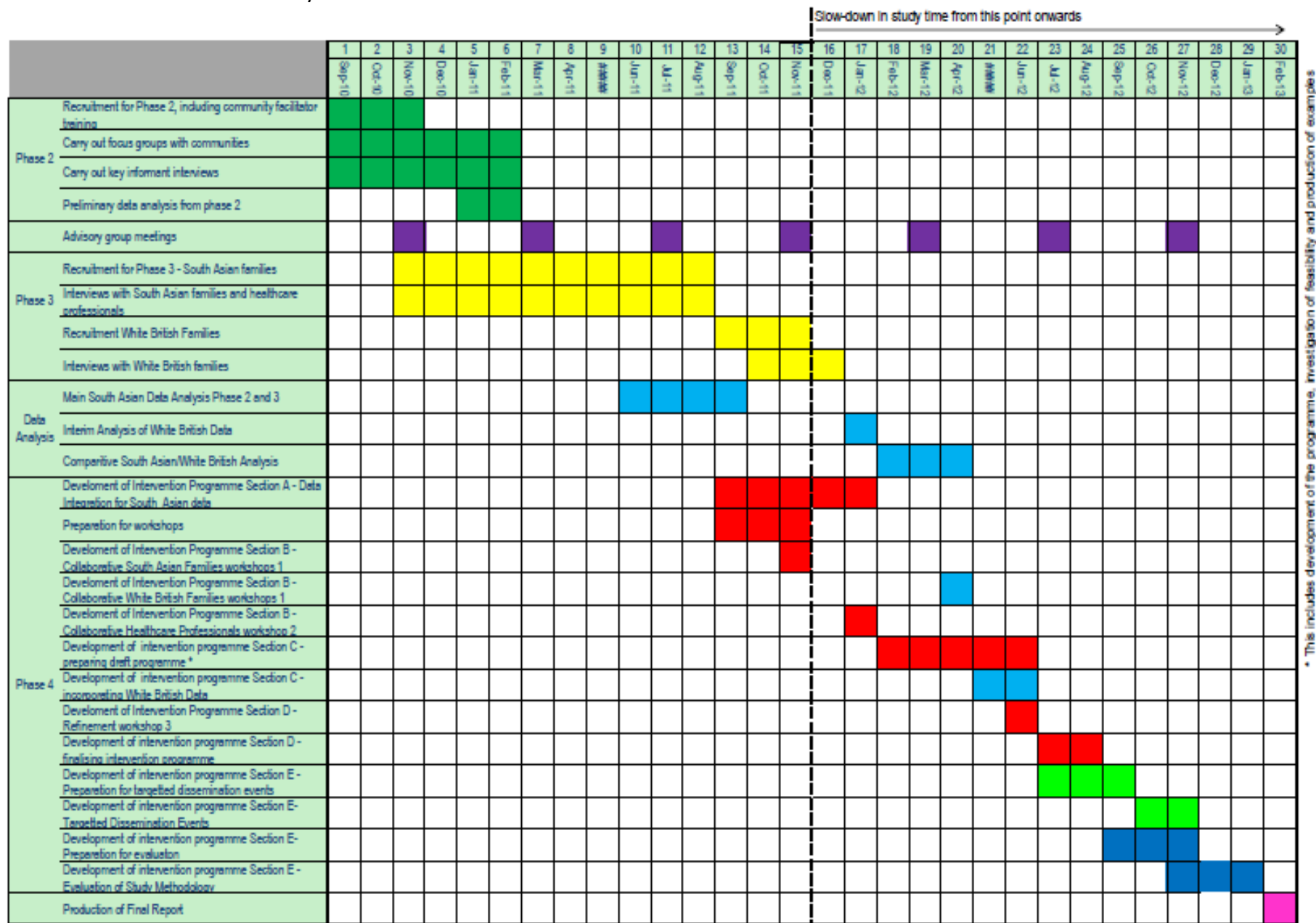

Supplement: Supplementary file 4 — Additional file 4. Timeline for Study - Gantt Chart. Gantt chart. (PDF 94 kb) [file 12874_2020_1148_MOESM4_ESM.pdf]
